# Nasal Sensitization with Ragweed Pollen Induces Local-Allergic-Rhinitis-Like Symptoms in Mice

**DOI:** 10.1371/journal.pone.0103540

**Published:** 2014-08-13

**Authors:** Yukinori Kato, Shoko Akasaki, Yoko Muto-Haenuki, Shigeharu Fujieda, Kazufumi Matsushita, Tomohiro Yoshimoto

**Affiliations:** 1 Laboratory of Allergic Diseases, Institute for Advanced Medical Sciences, Hyogo College of Medicine, Hyogo, Japan; 2 Department of Otorhinolaryngology-Head and Neck Surgery, Faculty of Medical Science, University of Fukui, Fukui, Japan; 3 Department of Otolaryngology-Head and Neck Surgery, Kyoto Prefectural University of Medicine, Kyoto, Japan; 4 Department of Immunology and Medical Zoology, Hyogo College of Medicine, Hyogo, Japan; Université Libre de Bruxelles, Belgium

## Abstract

Recently, the concept of local allergic rhinitis (LAR) was established, namely rhinitis symptoms with local IgE production and negative serum antigen-specific IgE. However, the natural course of LAR development and the disease pathogenesis is poorly understood. This study investigated the pathophysiology of mice with allergic rhinitis that initially sensitized with ragweed pollen through the nasal route. Mice were nasally administrated ragweed pollen over consecutive days without prior systemic immunization of the allergen. Serial nasal sensitization of ragweed pollen induced an allergen-specific increase in sneezing, eosinophilic infiltration, and the production of local IgE by day 7, but serum antigen-specific IgE was not detected. Th2 cells accumulated in nose and cervical lymph nodes as early as day 3. These symptoms are characteristic of human LAR. Continual nasal exposure of ragweed pollen for 3 weeks resulted in the onset of classical AR with systemic atopy and adversely affected lung inflammation when the allergen was instilled into the lung. *Fcer1a* ^−/−^ mice were defective in sneezing but developed normal eosinophilic infiltration. Contrary, *Rag2* ^−/−^ mice were defective in both sneezing and eosinophilic infiltration, suggesting that T cells play a central role in the pathogenesis of the disease. These observations demonstrate nasal allergen sensitization to non-atopic individuals can induce LAR. Because local Th2 cell accumulation is the first sign and Th2 cells have a central role in the disease, a T-cell-based approach may aid the diagnosis and treatment of LAR.

## Introduction

Allergic rhinitis (AR) is an important health problem because of its prevalence, impact on patient quality of life, and association with asthma. People worldwide potentially suffer from AR regardless of race, gender, or age. A study estimated that over 500 million people suffer from AR worldwide and that the incidence is increasing [1].

AR is induced by nasal allergen exposure that results in IgE-mediated inflammation of membranes lining the nose [2]. The allergic response in AR is divided into two phases: early and late [3]–[5]. In sensitized individuals, allergen-specific IgE is bound to FcεRI on the membrane of mast cells and basophils [3]. When they are crosslinked by cognate allergen, mast cells immediately secrete a preformed mediator, histamine, which induces early-phase responses: sneezing, itching and watery nasal discharge [3]–[5]. Early-phase responses occur within minutes after allergen exposure, while late-phase responses are evoked 6–10 h later. Inflammatory cell, e.g. eosinopihls, infiltration into nose mediates the major pathogenic change in the late-phase response. Eosinophils-derived mediators induce epithelial damage resulting in nasal mucosal swelling [3]–[5].

A diagnosis of AR is mainly based on nasal symptoms, sneezing and watery nasal discharge, and the detection of serum antigen-specific IgE [2], [5], [6]. Generally, patients with AR show increased serum total and antigen-specific IgE levels and a positive response to the skin prick test [2], [5], [6]. Patients who manifest symptoms similar to AR during the allergen season but do not show elevated serum IgE levels are classified as non-allergic rhinitis (NAR) [2], [5], [6]. However, studies have shown that within NAR patients, some are indeed “allergic” and have locally increased IgE levels in their nose or nasal lavage [7]–[9]. This type of rhinitis with local IgE production is defined as local allergic rhinitis (LAR) [7] or entopy [8], [9]. LAR is characterized by the local production of IgE in the nose, a Th2 pattern of mucosal cell infiltration, and positive nasal provocation test (NAPT), although serum antigen-specific IgE is negative [7]. A study conducted in Spain reported that within rhinitis patients in the study group, LAR was diagnosed in 25.7%, whereas NAR was 11.2% [10]. Importantly, LAR patients potentially evolve to systemic atopy and comorbid life-threatening allergic diseases, such as asthma, in later life [9], [11]. Thus, accurate diagnosis and correct treatment during early life is essential. However, because of a lack of both animal models and human studies, the pathogenesis of LAR is largely unknown.

AR pathogenesis has been well analyzed using murine models [12]–[14]. Most mouse studies of AR are performed by systemic sensitization of mice with an allergen and then later nasal challenge with the same allergen. Thus, they can be interpreted as models for patients sensitized with an allergen in early life. Although sensitization can occur through several tissues other than nose, such as skin, intestine, or lung, the initial sensitization in some patients is by the nasal route [3], [15]. However, how nasal sensitization occurs and immune response progression has not been investigated.

Here, we established a novel murine model of AR by serial nasal sensitization of ragweed pollen, a major airborne allergen in Central Europe and North America [16], without prior immunization of the allergen. Nasally sensitized AR mice developed symptoms closely resembling human LAR patients: allergen-specific sneezing, local IgE production, and negative for serum antigen-specific IgE. This study provides important implications for the diagnosis and treatment of LAR.

## Results

### Nasal sensitization of mice with ragweed mimics the symptoms of human LAR

**Figure 1:**
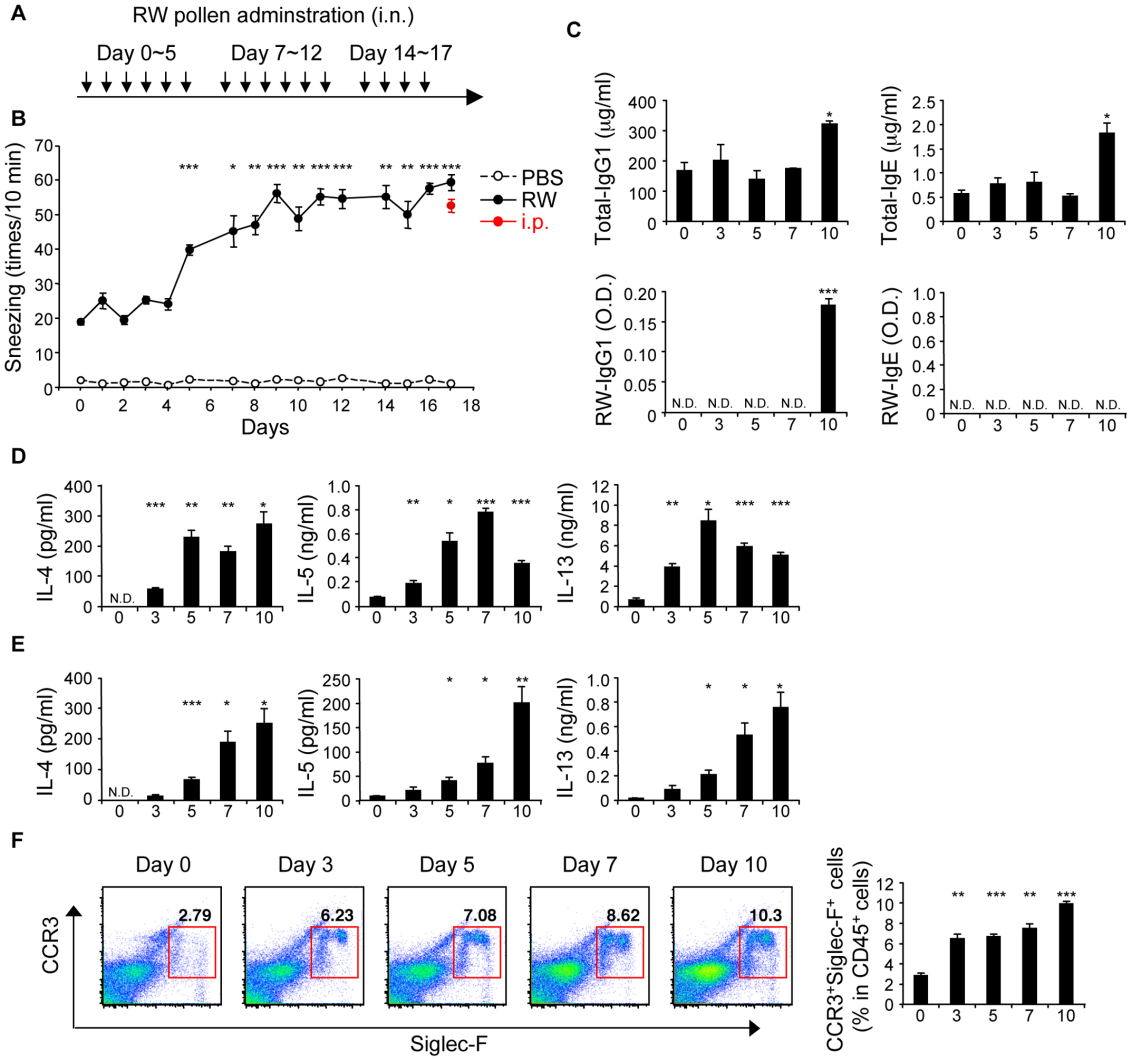
Nasal sensitization of mice with ragweed pollen mimics the symptoms of human local allergic rhinitis. Mice were nasally sensitized with ragweed (RW) pollen. (A) Experimental schema. (B) Numbers of sneezes. (C–F) Mice were nasally administered RW pollen on indicated consecutive days. (C) Serum immunoglobulin levels. Production of cytokines in cLN cells (D) and nasal CD4^+^ T cells (E). (F) Presence of eosinophils in nasal mucosa. Data representative of three independent experiments (means and SEMs of (B) five mice and (C–F) three mice). *P<0.05, **P<0.01, ***P<0.001. N.D. not detected.

To investigate the pathophysiology of patients who were initially sensitized with allergen through the nasal route, we established a novel murine model of AR without systemic immunization (Figure 1A; and Materials and Methods). Repeated nasal sensitization with ragweed induced an increase in sneezing over time (Figure 1B). The frequency of sneezing at later time points was comparable to systemically sensitized mice, an AR and systemic atopy model (Figure 1B see i.p.) [12]. The frequency of sneezing in ragweed-sensitized mice started to increase as early as day 5. However, serum total-IgG1 and IgE, and ragweed-specific IgG1 increased from day 10 and ragweed-specific IgE was not detected even at day 10 (Figure 1C). Although allergen non-specific nasal irritability could induce sneezing [17], heat-denatured ragweed administration failed to induce increased sneezing at day 7 (Figure S1). Thus, the antigen-specific response is essential for the increased frequency of sneezing in this model. The production of Th2 cytokines, IL-4, IL-5, and IL-13, from cervical lymph node (cLN) cells (Figure 1D) and CD4^+^ T cells residing in nasal mucosa (Figure 1E) by *in vitro* re-stimulation with ragweed extract significantly increased as early as day 3 or 5, respectively. cLN cells (Figure S2A) and nasal CD4^+^ T cells (Figure S2B) produced increased amounts of IL-5 and IL-13 in response to IL-33 in the absence of antigen stimulation at day 3, indicating the accumulation of IL-33-responsive Th2 cells in nasal mucosa. As for Th2 accumulation, CCR3^+^SiglecF^+^ eosinophils were recruited in nasal tissue as early as day 3 (Figure 1F). Thus, the development of allergic nasal symptoms occurs before the increase of serum IgE.

### Local IgE production by nasal ragweed sensitization

**Figure 2:**
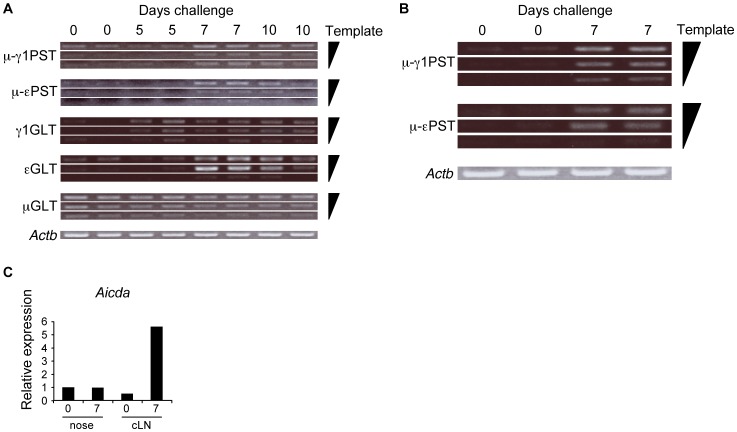
Local IgE production by nasal ragweed sensitization. Mice were nasally administered ragweed (RW) pollen on indicated consecutive days. GLTs and PSTs expression in cLN (A) and nasal (B) B cells. (C) qPCR analysis of *Aicda* expression in cLN and nasal B cells. Data representative of three independent experiments (n = 2). Nasal B cells were pooled from five mice.

To determine local IgE production in mice nasally sensitized with ragweed, the expression of ε and γ1 germ line transcript (GLT) and immunoglobulin mature post-switch transcript (PST) in cLN or nasal B cells was determined. Compared with naive mice, B cells from ragweed-sensitized mice expressed higher levels of μ-γ1 and μ-εPST, as well as γ1 and εGLT in cLN at days 7 and 10 (Figure 2A). In addition, nasal B cells from day 7 ragweed-sensitized mice showed expression of μ-γ1 and μ-εPST, while γ1 and εGLT was not detected from nasal B cells (Figure 2B and data not shown). mRNA expression levels of activation-induced cytidine deaminase (AID) were elevated in cLN, but not in nasal, B cells in day 7 ragweed-sensitized mice (Figure 2C). Thus, nasal ragweed-sensitization induced cLN B cells to undergo class-switch recombination (CSR) and differentiation into IgE-producing plasma cells. Although CSR was not detected in nasal tissues, IgE producing B cells accumulated by day 7 of ragweed-sensitization, when mice developed rhinitis symptoms yet serum antigen-specific IgE was negative.

Taken together, the novel murine model established here develops symptoms closely resembling human LAR patients [7]. Next, to investigate whether the LAR-like status of the mice is stable for a while, mice nasally sensitized with ragweed or PBS for 7 consecutive days were left untreated for next 7 days, then the mice were nasally challenged with ragweed for 3 days (day 14 to 16) (Figure S3A). Mice previously sensitized with ragweed showed increased sneezing even after a week break (Figure S3B). Of note, the serum ragweed-specific IgE was kept undetectable (Figure S3C). Thus, LAR-like status can persist for a while in mice even without repeated antigen exposure, and because the half-life of IgE in sera is extremely low compared to that in a tissue [18], IgE present in nose rather than the circulation may play a central role in inducing nasal symptoms.

### Repeated nasal ragweed sensitization induces systemic atopy

**Figure 3:**
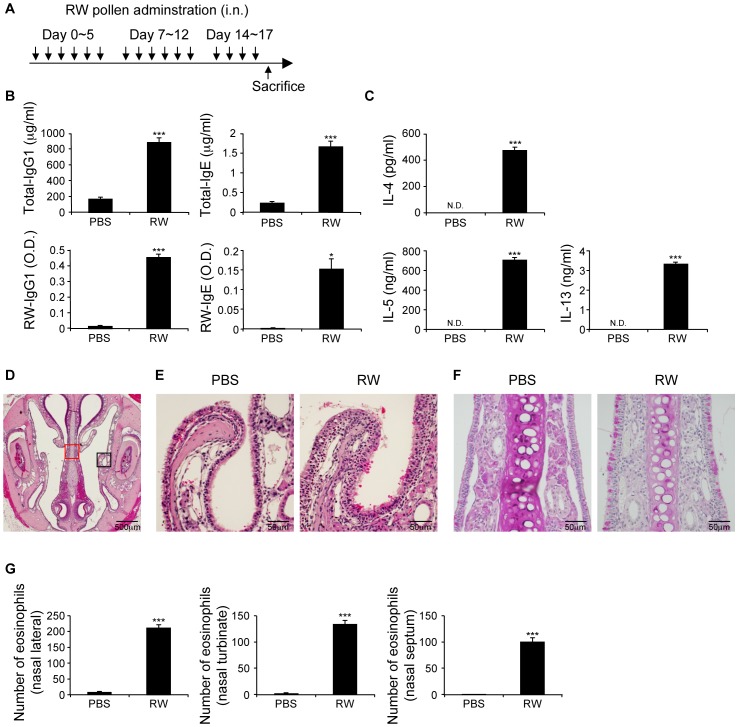
Repeated nasal ragweed sensitization induces systemic atopy. Nasal, ragweed (RW)-sensitized mice were analyzed 1 day after final sensitization. (A) Experimental schema. (B) Serum immunoglobulin levels. (C) Production of cytokines in cLN cells. (D–F) Histological examinations. (D) Hematoxylin and eosin (HE) staining of coronal section of nasal cavity. (E) HE staining of nasal lateral mucosa; black square in (D). (F) Periodic acid-Schiff staining of nasal septum; red square in (D). (G) Presence of eosinophils in nasal mucosa. Data representative of three independent experiments (means, SEMs, n = 5). *P<0.05, ***P<0.001. N.D. not detected.

We next examined whether sequential nasal ragweed-sensitization induced systemic atopy. We analyzed in mice nasally sensitized with ragweed 1 day after 3 weeks of sensitization (Figure 3A). Compared with the PBS group, ragweed-sensitized mice showed significantly increased total and ragweed-specific IgE and IgG1 levels in the sera (Figure 3B). Furthermore, cLN cells from ragweed-sensitized mice showed significantly elevated ragweed-specific Th2 cytokine production (Figure 3C). Histological examination revealed a multilayered epithelium, goblet cell hyperplasia, and infiltration of eosinophils in nasal mucosa (Figure 3D–F). Similar to human AR and LAR patients [19]–[22], mice nasally sensitized with ragweed responded well to topical corticosteroid treatment, which improved sneezing symptoms (Figure S4A). However, this treatment did not prevent increased ragweed-specific IgE and IgG1 production at the endpoint (Figure S4B).

**Figure 4:**
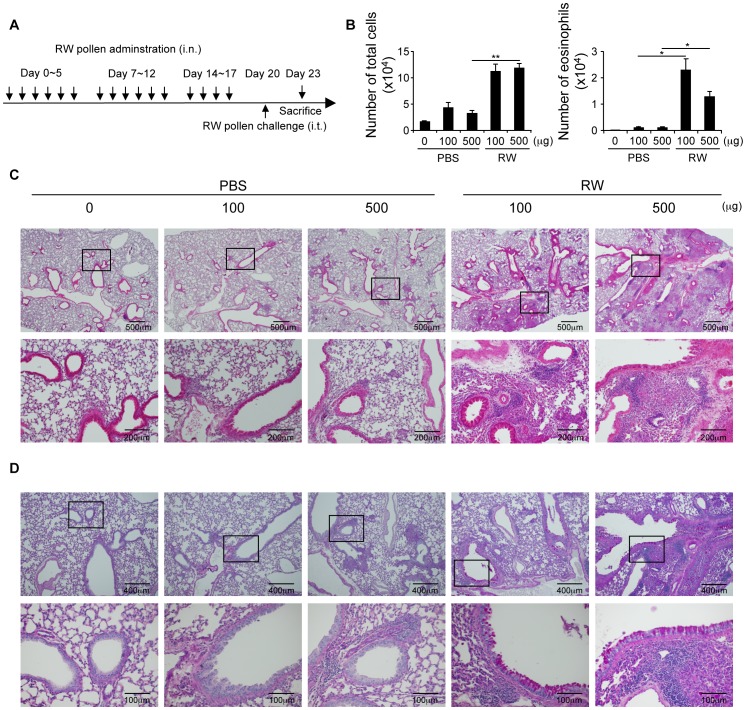
Nasal sensitization of ragweed adversely affects allergic inflammation in lungs. Nasal, ragweed (RW)-sensitized mice were intratracheally challenged with ragweed 3 days after final sensitization. (A) Experimental schema. (B) BALF CD45^+^ cells and eosinophils. Hematoxylin and eosin (C) and Periodic acid-Schiff (D) staining of lungs. Data representative of three independent experiments (means and SEMs, n = 4). *P<0.05, **P<0.01.

We next examined whether nasal allergen sensitization affected airway inflammation when the same allergen is instilled into the lung. In mice nasally sensitized with ragweed that were intratracheally challenged with ragweed (Figure 4A), there was a marked increase in inflammatory cells including eosinophils in the BALF compared with the non-sensitized group (Figure 4B). Furthermore, histological examination of lung specimens revealed severe cellular infiltration and goblet cell hyperplasia in the lungs of mice nasally sensitized with ragweed (Figure 4C and D).

These results demonstrated that nasal sensitization with an allergen induced systemic atopy, which can adversely affect the onset of other allergic diseases when the individuals encounter the same allergen.

### IgE has a central role in sneezing but is not essential for inflammatory cell accumulation in nasally sensitized AR

**Figure 5:**
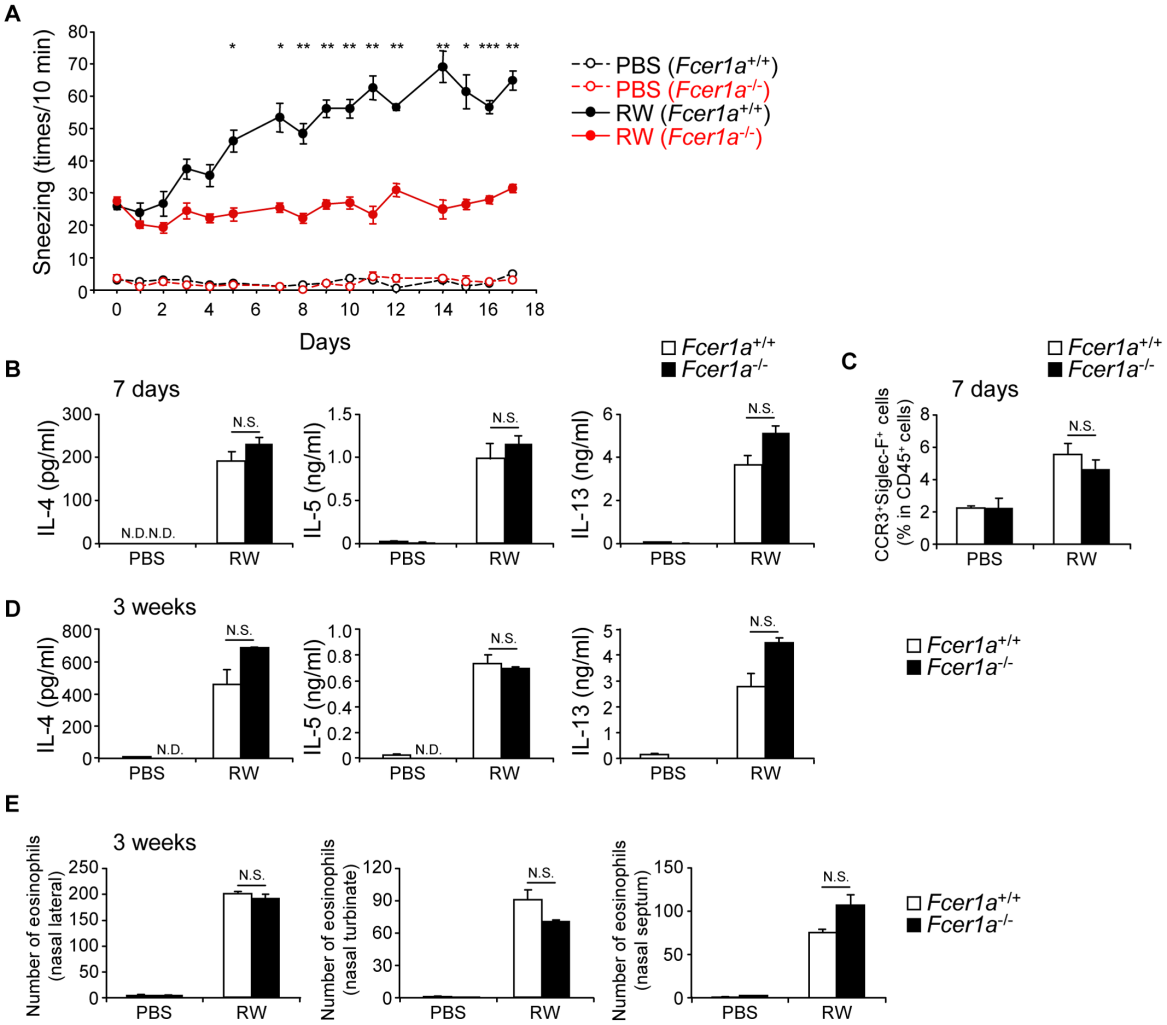
IgE-mediated activation of mast cells and basophils induce sneezing. WT and *Fcer1a* ^−/−^ mice were nasally administered ragweed (RW) pollen or PBS for 3 weeks as in Figure 3A. (A, D, E) or 7 consecutive days (B, C). (A) Number of sneezes. (B, D) Production of cytokines in cLN cells. (C, E) Presence of eosinophils in nasal mucosa. Data representative of two independent experiments (means, SEMs, n = 4). *P<0.05, **P<0.01, ***P<0.001. N.D. not detected. N.S. not significant.

Sneezing in AR is evoked by allergen-specific cross-linking of IgE on mast cells and basophils [3], [5], thus we measured this using a high-affinity receptor for IgE-deficient *Fcer1a* ^−/−^ mice and B-cell-deficient *lghm* ^−/−^ (μMT) mice. In both ragweed-sensitized *Fcer1a* ^−/−^ (Figure 5A) and μMT (Figure S5) mice, the elevation of sneezing was almost completely abrogated. In contrast, Th2 cytokine production from cLN cells and eosinophilic infiltration into the nose of ragweed-sensitized *Fcer1a* ^−/−^ mice were comparable to those in ragweed-sensitized WT mice at both early (7 days, Figure 5B and C) and late time points (3 weeks, Figure 5D and E). These results clearly show that IgE mediated signaling is essential for inducing sneezing, but is not sufficient for recruitment and activation of inflammatory cells at both early and late time points.

### Acquired immunity plays a central role in the pathogenesis of nasally sensitized AR

**Figure 6:**
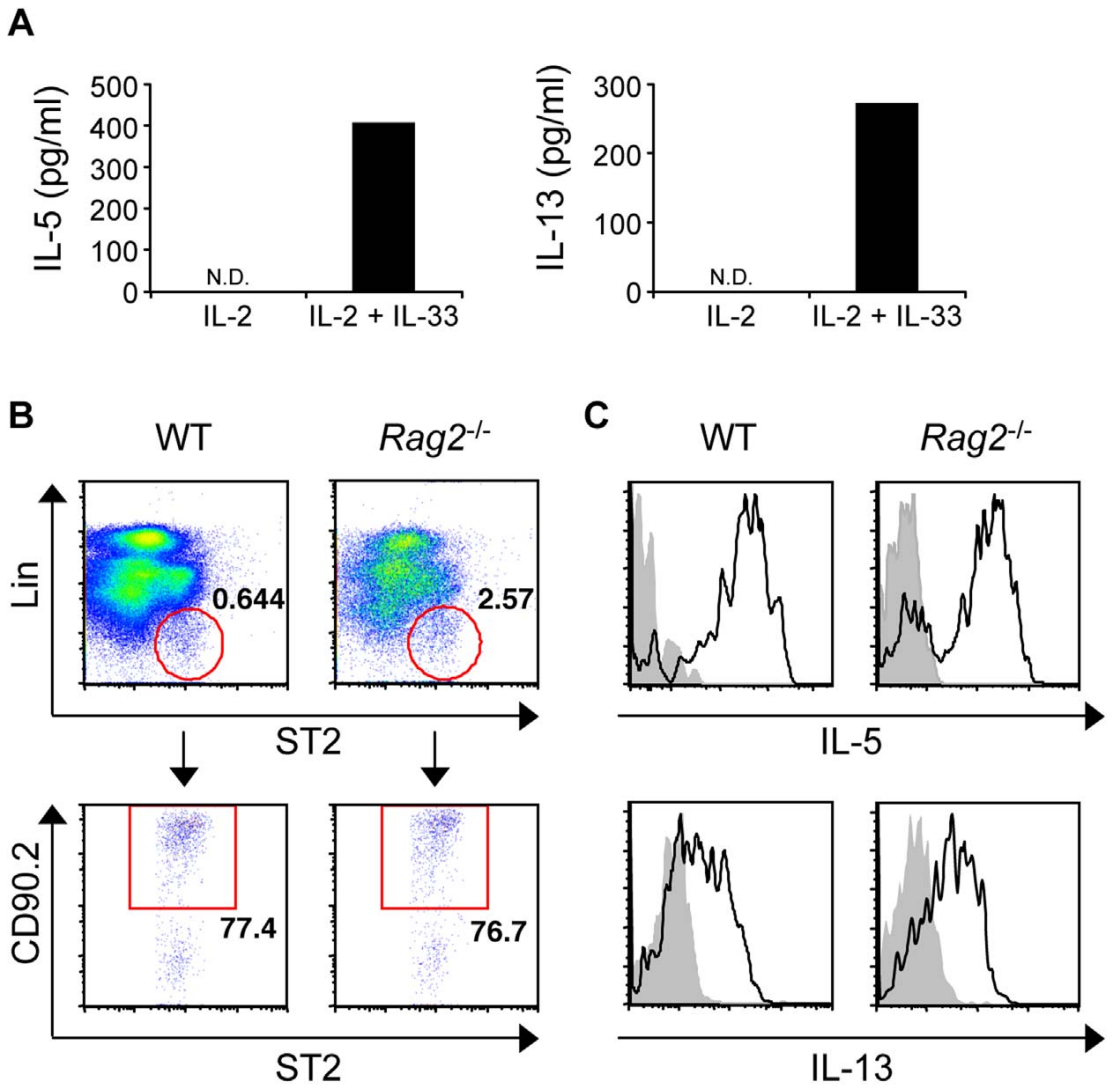
Nasal presence of group 2 innate lymphoid cells. (A) IL-5 and IL-13 from nasal CD3^−^ cells in response to IL-2 and IL-33. (B) Analysis and (C) IL-5 and IL-13 production of nasal Lin^−^ST2^+^CD90.2^+^ ILC2s from WT and *Rag2* ^−/−^ mice stimulated with PMA and ionomycin (black), isotype-matched control antibody (gray). Data representative of three independent experiments. N.D. not detected.

**Figure 7:**
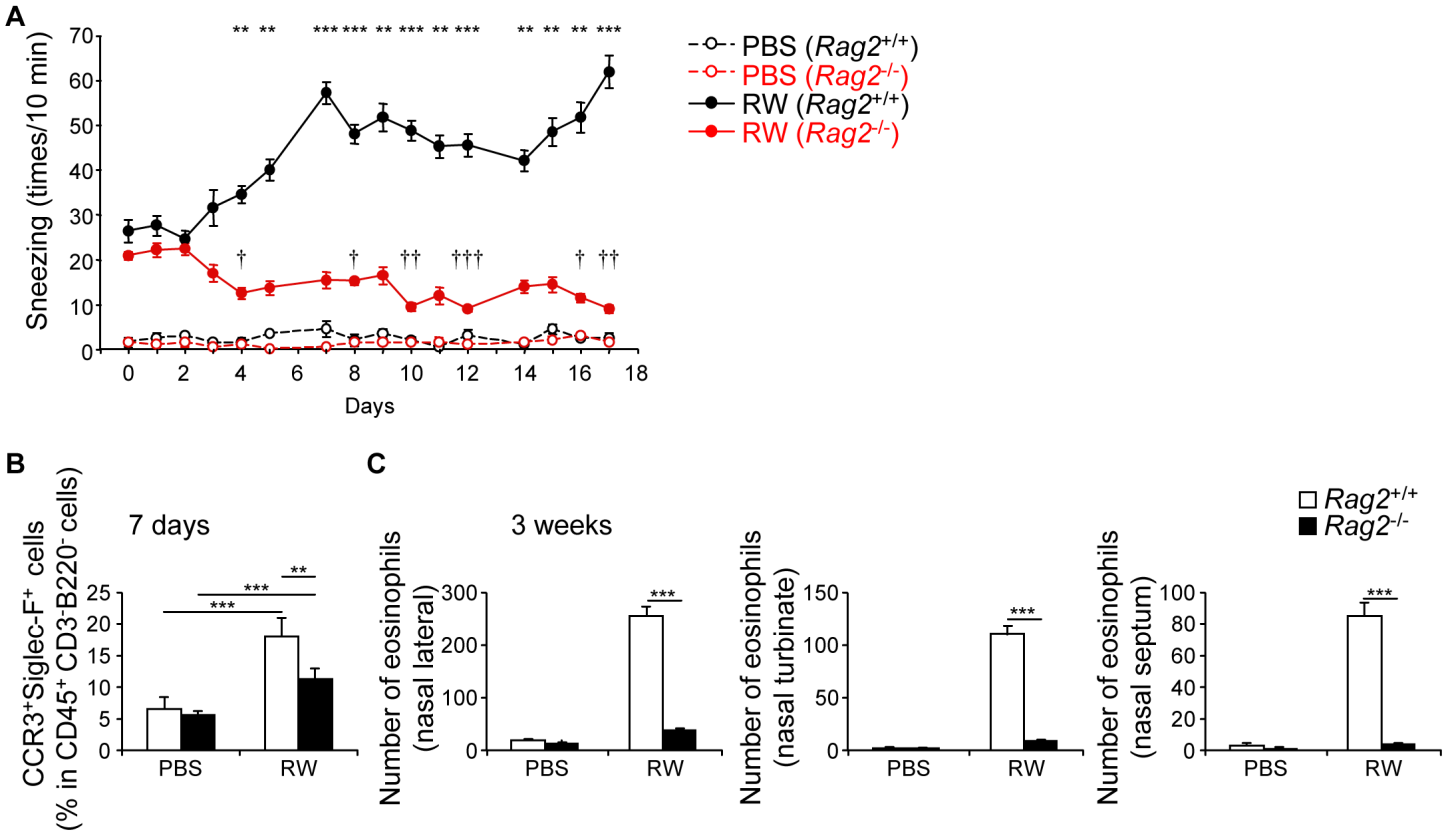
Acquired immunity is central for pathogenesis of nasally sensitized allergic rhinitis. WT and *Rag2* ^−/−^ mice were nasally administered ragweed (RW) pollen or PBS as in Figure 3A. (A, C) or for 7 consecutive days (B). (A) Number of sneezes. (B, C) Presence of eosinophils in nasal mucosa. Data representative of three independent experiments (means, SEMs n = 4). **P<0.01, ***P<0.001 (in (A), comparing WT to *Rag2* ^−/−^ mice). ^†^P<0.05, ^††^P<0.01, ^†††^P<0.001 (in (A), comparing to day 0 in *Rag2* ^−/−^ mice).

Recent studies have reported an essential role of group 2 innate lymphoid cells (ILC2s), characterized by the surface expression of ST2 (IL-33 receptor) and CD90 but negative for lineage markers, in allergic inflammation [23]–[25]. Indeed, CD3^−^ non-T-cell population in the nose produced IL-5 and IL-13 in response to IL-33 (Figure 6A). Furthermore, we identified a subset of ILC2-like cells in the nose (Figure 6B) that produced IL-5 and IL-13 in response to phorbol 12-myristate 13-acetate (PMA) and ionomycin (Figure 6C). Thus, to investigate the contribution of innate immunity to nasally sensitized AR, we used T- and B-cell-deficient *Rag2* ^−/−^ mice. In *Rag2* ^−/−^ mice, the frequency of sneezing was abrogated and rather significantly decreased over time, suggesting the T-cell-mediated maintenance of mast cell and/or basophil functions (Figure 7A). Eosinophilic infiltration in ragweed-sensitized *Rag2* ^−/−^ mice was significantly reduced at an early time point (7 days, Figure 7B), and completely abrogated at a late time point (3 weeks, Figure 7C). Although infiltration was reduced, ragweed-sensitized *Rag2* ^−/−^ mice still showed a significant influx of eosinophils into the nose compared with the PBS group at an early time point (Figure 7B). Thus, ILC2 might have a cooperative role in eosinophilic infiltration. However, acquired immunity has a central role in the pathogenesis of LAR and is the main therapeutic target for the disease.

## Discussion

In this study, we established a novel murine model of AR by serial nasal sensitization of ragweed without inducing prior systemic atopy and demonstrated several new findings. First, mice nasally sensitized with ragweed developed symptoms closely resembling human LAR. Second, mice repeatedly exposed to the allergen through the nasal route evolved systemic atopy. Third, local Th2 cell accumulation is the first sign of the disease. Fourth, IgE-mediated activation of mast cells and basophils is essential for inducing sneezing, but not involved in eosinophilic infiltration. Fifth, acquired immunity has a central role in the pathogenesis of LAR.

Mice nasally sensitized with ragweed had increased sneezing frequency, nasal infiltration of eosinophils, and local IgE production, before the elevation of serum IgE levels, characteristic of human LAR [7]. Thus, some LAR cases may be caused by the nasal sensitization of an allergen to individuals who do not have an atopy. Our mouse model here can be a useful tool for studying the pathophysiology of LAR.

Whether LAR patients potentially evolve to systemic atopy in later life remains a controversial subject. According to a report, sensitization to aeroallergens, not present at initial evaluation, were detected at the second examination 3–7 years later by means of a skin prick test, serum specific IgE measurements, or both in 24% of NAR patients [11]. This implied a conversion of LAR to AR. More recently, however, the same group reported that LAR patients and healthy controls evolved to systemic allergy in 6.81% and 4.5% cases, respectively, in a 5-year follow-up study [26]. They concluded that the difference was not significant and that LAR and AR are different entities. In our study, however, repeated nasal ragweed sensitization for 3 weeks in mice induced systemic atopy; increased serum total and ragweed-specific IgE levels. Furthermore, nasal sensitization with an allergen adversely affected airway inflammation when the same allergen was instilled into the lung, suggesting LAR evolves to classic AR and is associated with other allergic inflammatory diseases. Thus, larger scale and longer period human studies are essential to conclude convincingly a relationship between LAR and AR. Several reports demonstrated that treatment of patients with LAR or AR with topical nasal corticosteroids improved nasal symptoms [19]–[22]. Indeed, our study also showed that topical nasal corticosteroid treatment of ragweed-sensitized mice dramatically reduced sneezing frequency. Because corticosteroid-treated mice showed increased total and ragweed-specific IgE and IgG1 sera levels comparable to untreated mice, treatment does not prevent the development of systemic atopy. Therefore, it is essential to diagnose LAR precisely and as early as possible to avoid further antigen sensitization.

Recently, a diagnostic flow-chart for LAR was proposed: the first step is the skin prick test or systemic antigen-specific IgE (diagnosis of AR), the second is nasal IgE or NAPT (diagnosis of LAR), and the third is the atopy patch test (diagnosis of T-cell-mediated allergy) [7], [27], [28]. Interestingly, our mouse model developed similar symptoms over time in reverse order (Th2 cells, nasal IgE, and then systemic antigen-specific IgE). Thus, LAR and AR can occur during the natural course of disease, and the first detectable sign could be the emergence of local antigen-specific Th2 cells. This implies that T-cell-based diagnosis is a promising approach for LAR. The local accumulation of Th2 cells increased over time and correlated well with rhinitis symptoms. As Th2 cells express high levels of ST2 [29], T cells accumulating in the nose after ragweed-sensitization responded well to *in vitro* IL-33 stimulation. Detecting local IL-33-responsive T cells could be a universal and easy way to identify the presence of Th2 cells.

Histamine released by the allergen specific cross-linking of IgE on mast cells and basophils is considered central for sneezing in AR [30]. Indeed, we demonstrated that increased sneezing induced by nasal sensitization with ragweed was mediated by B cells and IgE production. Thus, local IgE production in the nose has a crucial role in the early phase response of LAR and blocking local IgE may improve sneezing symptoms. However, the nasal sensitization of ragweed in mice showed the recruitment of eosinophils in the nose and activation of Th2 cells even in the absence of IgE-mediated signaling. IgE-mast cell pathways can induce eosinophil migration and activation mediated by the production of prostaglandins or leukotrienes [31]–[33]. However, our study suggests that activated Th2 cells, rather than B cells and IgE, play an essential role in eosinophil accumulation in the nose of nasally sensitized AR mice.

Recent studies reported ILC2s producing IL-5 and IL-13 in response to IL-25 and/or IL-33 have an essential role in allergic airway inflammation in the absence of adaptive immunity [23]–[25]. We found that ILC2s resided in the nose of naive mice and produced IL-5 and IL-13. However, although ILC2s may contribute to the early time points of eosinophilic infiltration in ragweed-sensitized mice, they are not involved in the late or chronic phase of eosinophilic infiltration in the nose. Rather, acquired immunity has an essential role in both early- and late-phase responses in nasally sensitized AR. Because *Rag2* ^−/−^, but not μMT, mice showed decreased sneezing over time, T cells may be important for maintaining mast cell and/or basophil functions. Thus, T-cell-targeted therapy might be effective for treating LAR. Because ILC2s can induce Th2-type inflammation without allergen specificity, it is interesting to explore the involvement of ILC2s in non-allergic or idiopathic rhinitis. Furthermore, although our data demonstrate that ILC2 alone cannot induce strong nasal responses, these cells may interact with mast cells, T cells [34] or DCs [35] and participate in inducing Th2-type immune responses in the nose. Further studies are required to clarify the role of ILC2s in allergic or non-allergic inflammation in the nose.

In summary, we established a novel murine model of AR by serial nasal sensitization with ragweed that develops symptoms closely resembling human LAR patients. Further studies using our mouse model might provide better diagnosis and therapy for LAR. Because the emergence of local Th2 cells is the first detectable sign and they have a central role in disease pathogenesis, T-cell-based diagnosis and therapy may improve LAR treatment.

## Materials and Methods

### Ethics Statement

All animal experiments were performed with approval of (No. A11-235 and No. 28028), and in accordance with the guidelines of, the Institutional Animal Care Committee of Hyogo College of Medicine, and every effort was made to minimize the suffering of the animals. The euthanasia of all experimental mice was achieved by cervical dislocation.

### Mice

Wild-type (WT) BALB/c mice were purchased from Charles River Laboratories Japan, Inc (Yokohama, Japan). BALB/c *Fce1a* ^−/−^ mice and *lghm* ^−/−^ (μMT) mice were purchased from Jackson Laboratories (Bar Harbor, ME). BALB/c *Rag2* ^−/−^ mice were purchased from Taconic (Germantown, NY).

### Reagents

Recombinant mouse IL-33 was purchased from Hokudo Co., Ltd (Sapporo, Japan). Fluorescent-labeled antibodies for CCR3 (83101) were purchased from R&D Systems (Minneapolis, MN). Antibodies for Siglec-F (E50-2440), CD45R/B220 (RA3-6B2), CD49b (DX5), CD11b (M1/70), CD11c (N418), and CD90.2 (53-2.1) were purchased from BD Biosciences (San Diego, CA). CD45 (30-F11), CD19 (6D5), CD3ε (145-2C11), IL-5 (TRFK5), CD16/32 (93), and streptavidin were purchased from BioLegend (San Diego, CA). Antibodies for IL-13 (eBio13A) and IgG1 isotype control (eBRG1) were purchased from eBioscience (San Diego, CA). Anti-IgE (23G3) was purchased from Southern Biotechnology Associates, Inc (Birmingham, AL). Biotinylated anti-T1/ST2 antibody was purchased from MD Biosciences (St Paul, MN). Ragweed pollen was purchased from PolyScience (Niles, IL). Ragweed extract was purchased from LSL Co Ltd (Tokyo, Japan). Anti–2,4-dinitrophenyl (DNP) IgE mAb (SPE-7) was purchased from Sigma-Aldrich (St Louis, MO). Purified mouse IgG1 λ isotype control was purchased from BD Biosciences.

### Mouse model of AR

Mice were nasally administered ragweed pollen, 1 mg in 20 µL of phosphate buffered saline (PBS) or 20 µL PBS alone for 6 consecutive days in the first and second week, and 4 consecutive days in the third week (days 0–5, 7–12, and 14–17) (Figure 1A and Figure 3A). The frequency of sneezing for 10 min immediately after every nasal challenge was determined. In some experiments, mice were nasally administered ragweed pollen (1 mg in 20 µL of PBS) for 3, 5, 7, or 10 consecutive days without a break. To induce lung inflammation, mice intranasally sensitized with ragweed pollen or PBS for 3 weeks were then further intratracheally challenged with ragweed pollen 3 days after the final nasal sensitization. Bronchoalveolar lavage fluid (BALF) and lungs were obtained 3 days after intratracheal challenge (Figure 4A).

### Cell preparation

Mice were sacrificed and noses and cervical lymph nodes (cLN) were dissected for further analysis. Noses were cut into small fragments with scissors and digested for 50 min at 37°C with collagenase (150 U/ml) and DNase I (10 µg/ml). Cell suspensions were filtered using a cell strainer and red blood cells were lysed. cLNs were dissected from mice, and single cell suspensions were prepared by sieving and gentle pipetting. CD4^+^ or B220^+^ cells from cLNs were sorted by AutoMACS Separator (Miltenyi Biotec, Bergisch Gladbach, Germany). Nasal CD45^+^B220^+^CD19^+^CD3ε^−^ cells were sorted by FACSAria I (BD Bioscience).

### *In vitro* cytokine production

cLN cells or nasal CD4^+^ T cells were isolated from mice and cultured for 5 days in 96-well plates at 2×10^5^ or 5×10^4^, respectively, per 0.2 mL per well with IL-2 (100 pmol/L) and ragweed extract (5 µg/mL) in the presence of 1×10^5^ irradiated conventional antigen-presenting cells (T cell–depleted BALB/c splenic cells) or stimulated with IL-2 (100 pmol/L) alone or IL-2 plus IL-33 (10 ng/mL) in RPMI-1640 (Sigma, St Louis, MO) supplemented with 10% (vol/vol) FBS, penicillin (100 U/mL), streptomycin (100 µg/mL), L-glutamine (2 mM), and β-mercaptoethanol (50 µM). The culture supernatants were collected and cytokine concentrations were assessed by enzyme-linked immunosorbent assay (ELISA).

### Flow cytometry

For surface staining, cells were washed in ice-cold staining buffer (1% BSA in PBS), then incubated with each antibody for 20 min and washed twice with staining buffer. Data were acquired on a FACSCanto II flow cytometer (BD Biosciences) and analyzed using FlowJo software (version 7.6.1, Tree Star Inc., Ashland, OR). For intracellular IL-5 or IL-13 staining, nasal cells were isolated and stimulated with PMA (50 ng/mL) and ionomycin (2.6 µg/mL) in the presence of GolgiStop for 4 hours. Intracellular staining was performed using BD Cytofix/Cytoperm Plus Fixation/permeabilization Kit (BD biosciences) according to the manufacturer's instructions. Group 2 innate lymphoid cells were defined as lineage marker (B220, CD3, CD11b, CD11c, CD49b, and FcεRI) negative and ST2 and CD90.2 positive cells.

### Histology

Histological examinations of mouse nose specimens were performed as previously described [12]. Briefly, facial skin was stripped, heads were severed between the upper and lower jaws, and noses were removed. Fixed in 4% paraformaldehyde for 3 days and decalcified in 0.12 mol/L EDTA solution (pH 6.5) for 7 days at room temperature. After decalcification, specimens were embedded in paraffin, and 4 µm coronal sections were sectioned and stained with hematoxylin and eosin (HE) or periodic acid–Schiff (PAS).

Left lobes of lungs were isolated and fixed in 4% paraformaldehyde for 1 day, embedded in paraffin, sectioned at 4 µm and stained with HE or PAS.

### Corticosteroid treatment of mice

For treatment with Fluticasone Furote (FF) (GlaxoSmithKine, Middlesex, UK), mice were intranasally administered FF every day from day 6 of RW-sensitization. For RW pollen administration, mice were administered FF 1 hour before RW administration.

### ELISA

Peripheral blood was collected from the inferior vena cava or heart 24 hours after the final nasal challenge. Total IgE and IgG1 levels were measured by using ELISA, as described previously [36]. Biotin-conjugated ragweed extract was prepared in our laboratory to detect ragweed-specific IgE and IgG1 in sera [37].

### Quantitative PCR analysis

Total RNAs from B220^+^ cLN or nasal cells were isolated using RNeasy MiniKit (Qiagen, Venlo, Netherlands) and cDNA was synthesized using ReverTra Ace (Toyobo, Osaka, Japan). The expression of genes was quantified with TaqMan Gene Expression Assays (Applied Biosystems) and Thermal cycler dice RT-PCR system (Takara Bio Inc., Otsu, Japan) according to the manufacturer's instructions. The results were shown as the relative expression normalized to the expression of a gene encoding eukaryotic 18S rRNA. The specific primers and probes used for quantitative RT-PCR were TaqMan probes for *Aicda* and 18S rRNA (Applied Biosystems).

### PCR

For PCR of GLTs and PSTs, the following primers were used according to Muramatsu et al. [38]: (μGLT) IμF and CμR, (γ1GLT) Iγ1 and Cγ1R, (εGLT) IεF and CεR, (μ-γ1PST) IμF and Cγ1R, and (μ-εPST) IμF and CεR. μ, γ1, εGLTs were amplified by 30, 35, and 37 cycles of PCR, respectively. μ-γ PLT and μ-ε PLT were amplified by 35 and 37 cycles of PCR, respectively. IμF: 5′-CTCTGGCCCTGCTTATTGTTG-3′. Iγ1F: 5′-GGCCCTTCCAGATCTTTGAG-3′. IεF: 5′-TGGGATCAGACGATGGAGAATAG-3′. CμR: 5′-GAAGACATTTGGGAAGGACTGACT-3′. Cγ1R: 5′-GGATCCAGAGTTCCAGGTCACT-3′. CεR: 5′-CCAGGGTCATGGAAGCAGTG-3′

### Bronchoalveolar lavage fluid

BALF was performed with 500 µl of PBS per right lobe of lung tissues. Total cells (CD45^+^) and eosinophils (CD45^+^CCR3^+^Siglec-F^+^CD11c^−^) were examined by flow cytometry analysis.

### Statistics

Statistical significance was calculated by the two-tailed Student's *t*-test. *P* values <0.05 were considered statistically significant. All experiments were performed two or three times and representative results are shown.

## Supporting Information

Figure S1 **Increase in sneezing frequency of mice nasally-administrated ragweed pollen is antigen-specific.** WT mice nasally administered ragweed (RW) pollen for 7 consecutive days were intranasally challenged with RW pollen or heat-denatured RW pollen (100°C incubation for 30 minutes) at day 7. Numbers indicate frequency of sneezing. Data are representative of three independent experiments (means, SEMs, n = 5). **P<0.01.
(TIF)

Figure S2 **Nasal CD4^+^ T cells produce significant amount of IL-5 and IL-13 in response to IL-33 in mice nasally sensitized to ragweed.** WT mice were nasally administered ragweed (RW) pollen for indicated consecutive days. Cytokine production by cLN cells (A) or nasal CD4^+^ T cells (B) stimulated with IL-2 alone or IL-2 plus IL-33 for 5 days. Data are representative of three independent experiments (means, SEMs, n = 3). *P<0.05, **P<0.01 and ***P<0.001. N.D. not detected.
(TIF)

Figure S3 **The local allergic rhinitis-like symptoms persist without repeated ragweed exposure.** Mice were nasally sensitized with ragweed (RW) pollen. (A) Experimental schema. (B) Numbers of sneezes. (C) Serum immunoglobulin levels. Data are representative of two independent experiments (means, SEMs, n = 5). *P<0.05 and ***P<0.001. N.D. not detected.
(TIF)

Figure S4 **Treatment of topical nasal corticosteroid in ragweed-sensitized mice ameliorates sneezing symptom.** WT mice were nasally administered ragweed (RW) pollen or PBS by experimental protocol as in Figure 1A. The corticosteroid (Fluticasone Furote; FF) was nasally applied every day from day 6. (A) Numbers indicate the frequency of sneezing. (B) Immunoglobulin levels in the sera at day 18. Data are representative of two independent experiments (means, SEMs, n = 5). *P<0.05 and **P<0.01. N.D. not detected. N.S. not significant.
(TIF)

Figure S5 **Sneezing symptoms are ameliorated in** ***lghm*** **^−/−^ mice.** WT and *lghm* ^−/−^ mice were nasally administered ragweed (RW) pollen or PBS by experimental protocol as in Figure 1A. Numbers indicate the frequency of sneezing. Data are representative of two independent experiments (means, SEMs, n = 4). *P<0.05.
(TIF)
