# Supplementary figures and images for: Nasal Sensitization with Ragweed Pollen Induces Local-Allergic-Rhinitis-Like Symptoms in Mice
Source: PLoS One. 2014 Aug 13;9(8):e103540. doi: 10.1371/journal.pone.0103540 (PMC4132107; doi:10.1371/journal.pone.0103540)

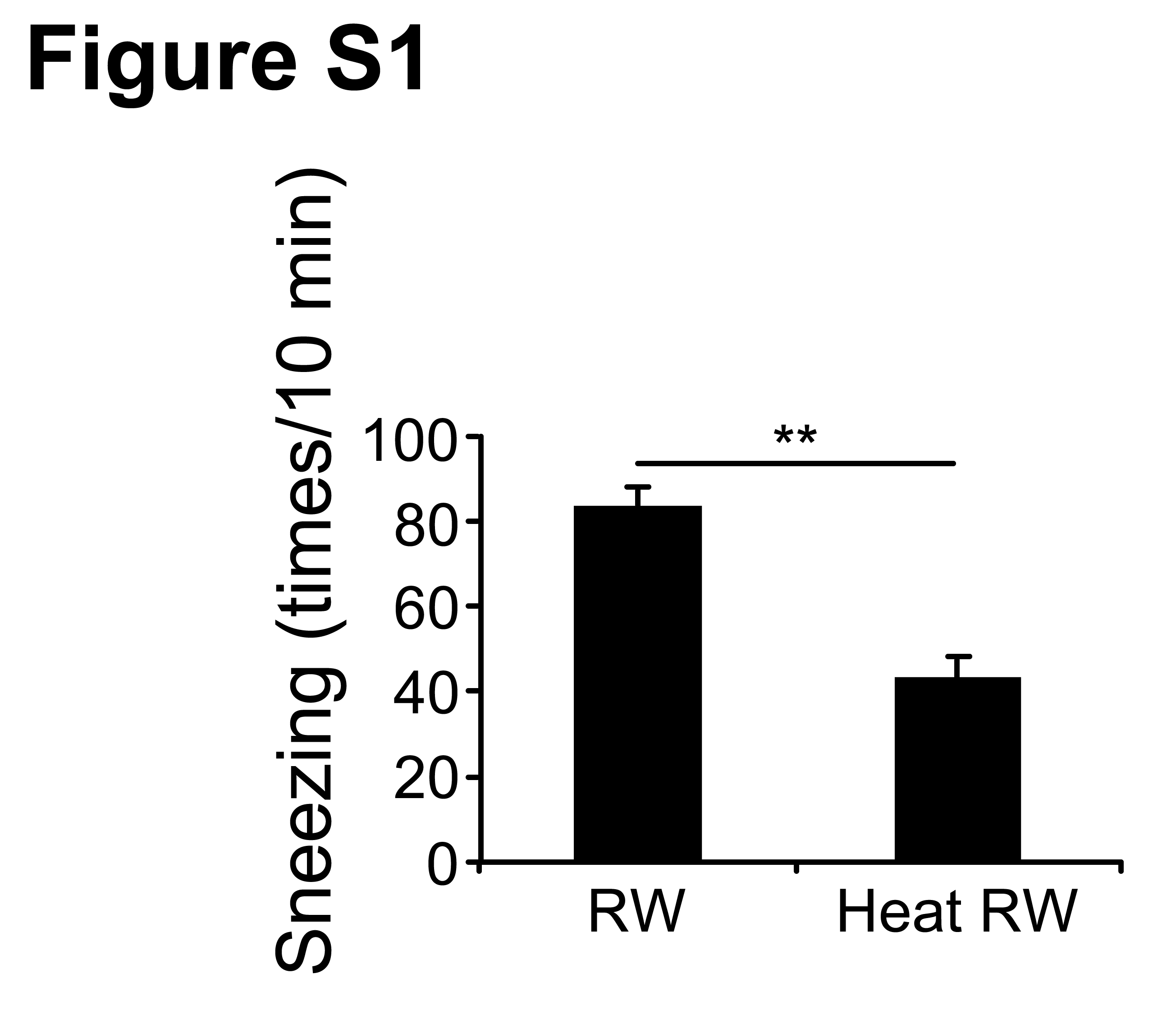

Supplement: Figure S1 — Increase in sneezing frequency of mice nasally-administrated ragweed pollen is antigen-specific. WT mice nasally administered ragweed (RW) pollen for 7 consecutive days were intranasally challenged with RW pollen or heat-denatured RW pollen (100°C incubation for 30 minutes) at day 7. Numbers indicate frequency of sneezing. Data are representative of three independent experiments (means, SEMs, n = 5). **P<0.01. (TIF) [file pone.0103540.s001.tif]

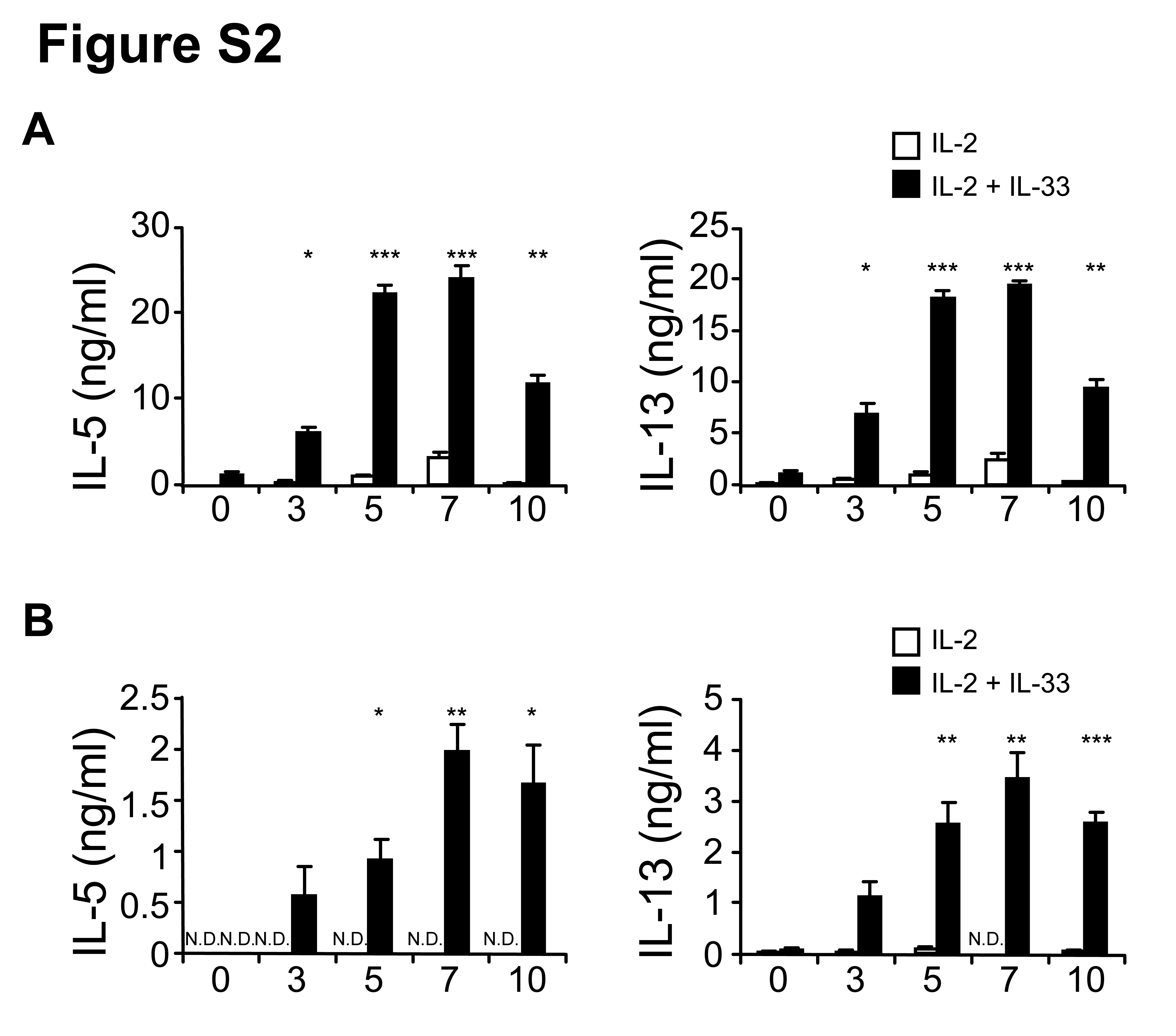

Supplement: Figure S2 — Nasal CD4+ T cells produce significant amount of IL-5 and IL-13 in response to IL-33 in mice nasally sensitized to ragweed. WT mice were nasally administered ragweed (RW) pollen for indicated consecutive days. Cytokine production by cLN cells (A) or nasal CD4+ T cells (B) stimulated with IL-2 alone or IL-2 plus IL-33 for 5 days. Data are representative of three independent experiments (means, SEMs, n = 3). *P<0.05, **P<0.01 and ***P<0.001. N.D. not detected. (TIF) [file pone.0103540.s002.tif]

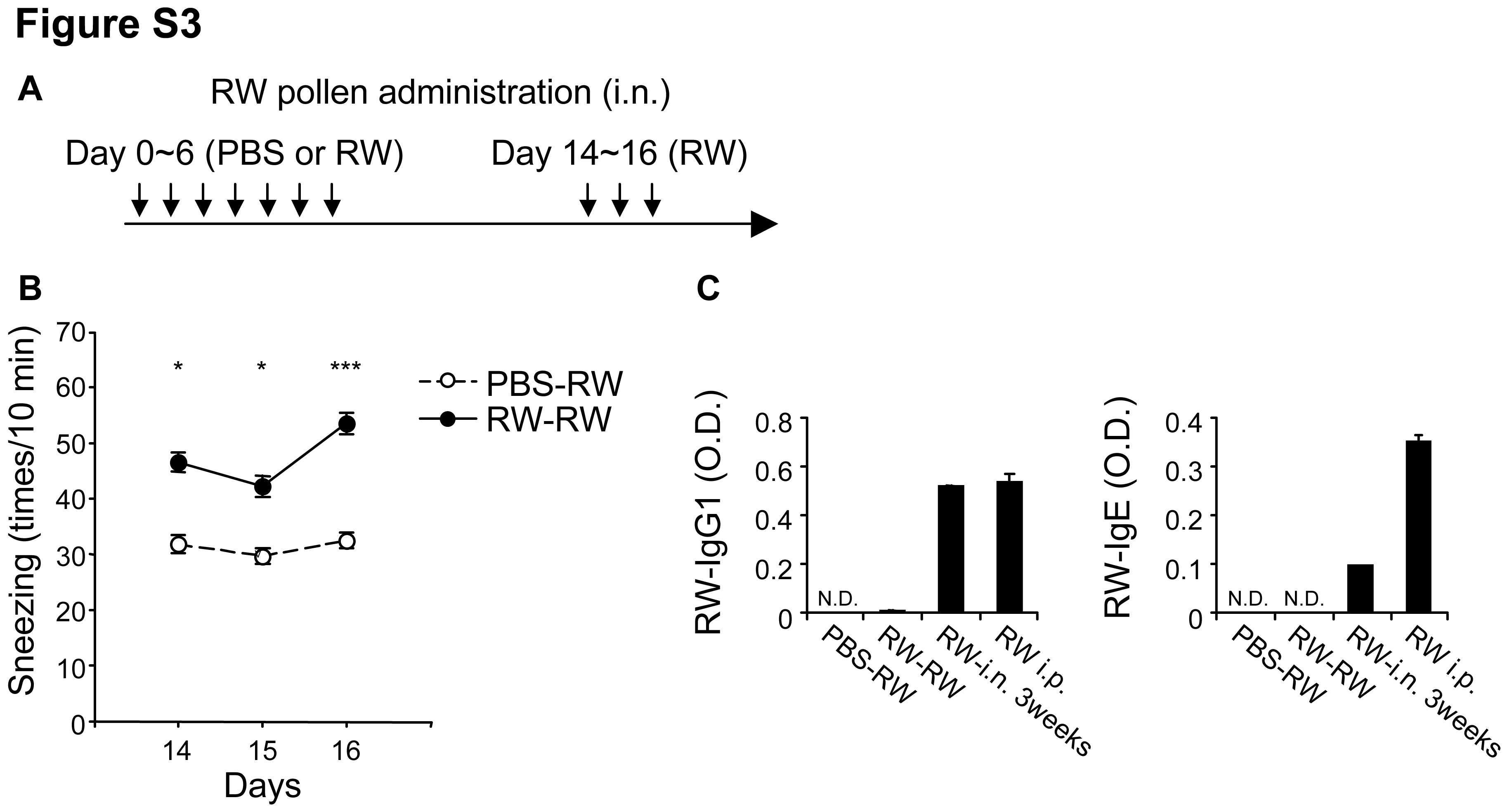

Supplement: Figure S3 — The local allergic rhinitis-like symptoms persist without repeated ragweed exposure. Mice were nasally sensitized with ragweed (RW) pollen. (A) Experimental schema. (B) Numbers of sneezes. (C) Serum immunoglobulin levels. Data are representative of two independent experiments (means, SEMs, n = 5). *P<0.05 and ***P<0.001. N.D. not detected. (TIF) [file pone.0103540.s003.tif]

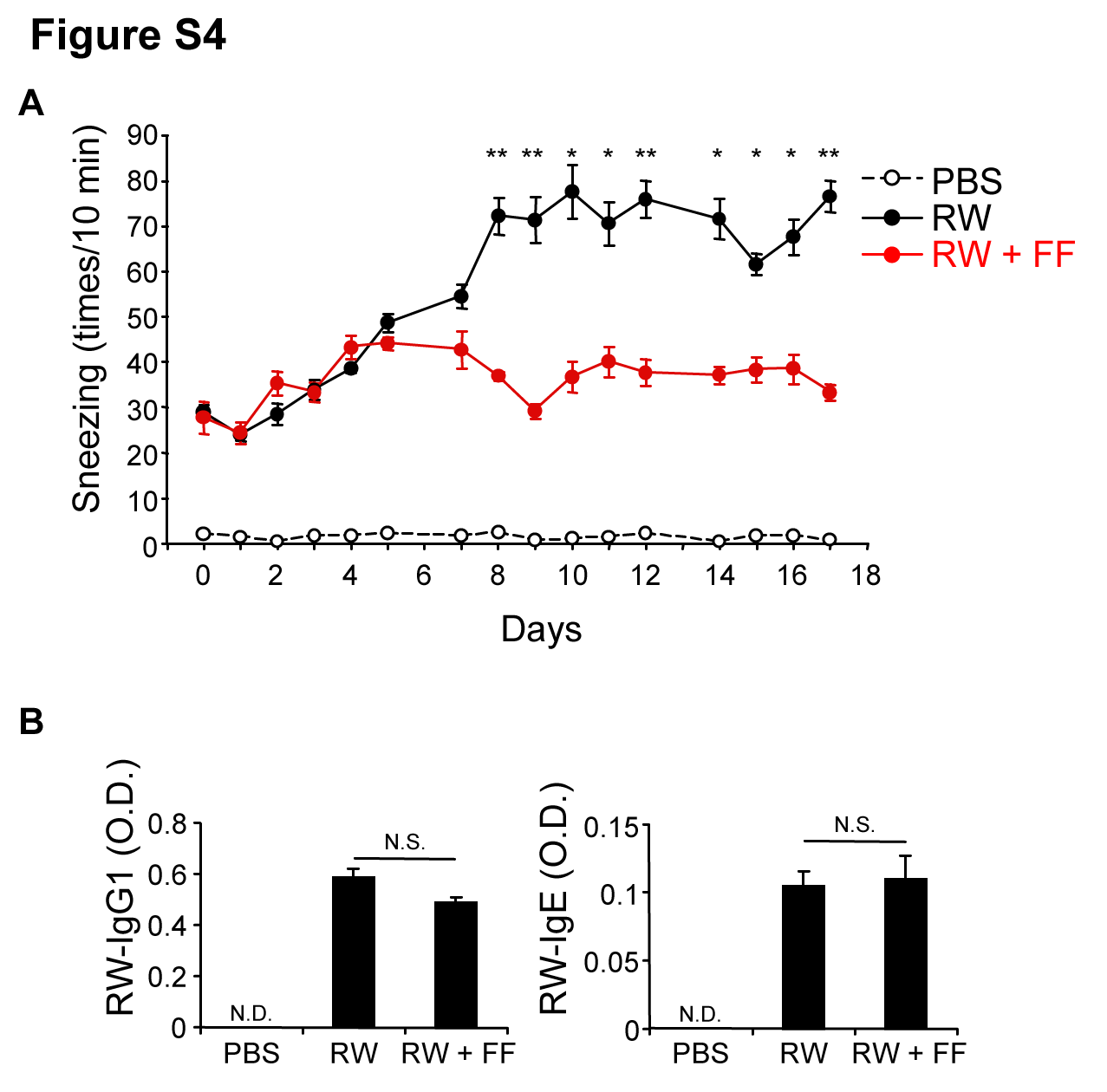

Supplement: Figure S4 — Treatment of topical nasal corticosteroid in ragweed-sensitized mice ameliorates sneezing symptom. WT mice were nasally administered ragweed (RW) pollen or PBS by experimental protocol as in Figure 1A. The corticosteroid (Fluticasone Furote; FF) was nasally applied every day from day 6. (A) Numbers indicate the frequency of sneezing. (B) Immunoglobulin levels in the sera at day 18. Data are representative of two independent experiments (means, SEMs, n = 5). *P<0.05 and **P<0.01. N.D. not detected. N.S. not significant. (TIF) [file pone.0103540.s004.tif]

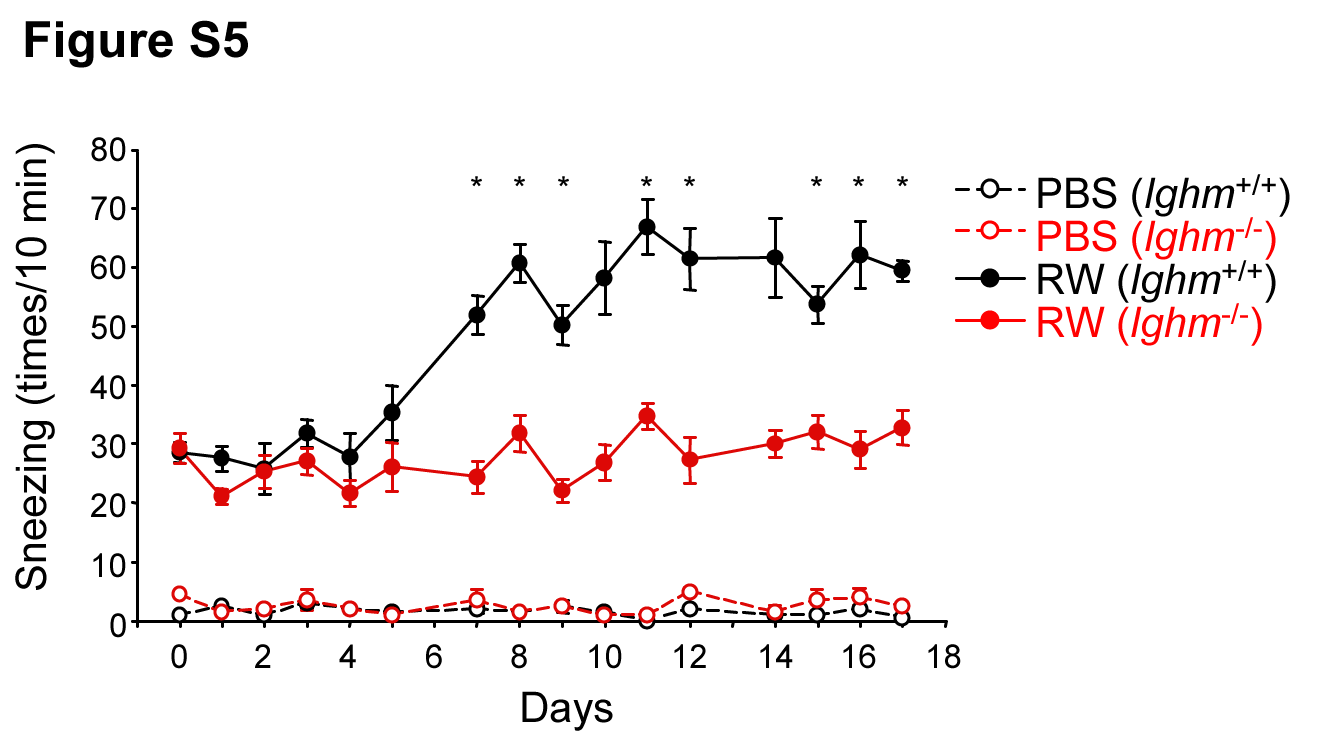

Supplement: Figure S5 — Sneezing symptoms are ameliorated in lghm −/− mice. WT and lghm −/− mice were nasally administered ragweed (RW) pollen or PBS by experimental protocol as in Figure 1A. Numbers indicate the frequency of sneezing. Data are representative of two independent experiments (means, SEMs, n = 4). *P<0.05. (TIF) [file pone.0103540.s005.tif]
